# Feasibility and Acceptability of mPallCare, a Digital Health Intervention for People Living With Advanced Cancer in a Refugee Settlement in Uganda: Mixed Methods Study

**DOI:** 10.2196/73483

**Published:** 2026-06-26

**Authors:** Eve Namisango, Agatha Aduro, William Goodman, Shaunna Burke, Raphael Ryabonye, Nickson Mutaasa, Timothy Muyami, Elizabeth Nabirye, Dennis Olodi, Viola Ederu, Bassey Ebenso, Omolola Salako, Kehinde Okunade, Desiree Azizoddin, Mhoira Leng, Karl Lorenz, Felix Muehlensiepen, Richard A Powell, Matthew Allsop

**Affiliations:** 1 Digital Health Hub, College of Medicine, University of Lagos, Lagos, Nigeria; 2 African Palliative Care Association, Kampala, Uganda; 3 Academic Unit of Palliative Care, Leeds Institute of Health Sciences, University of Leeds, Worsley Building, Leeds, LS2 9LU, United Kingdom, 44 1133434185; 4 School of Biological Sciences, University of Leeds, Leeds, United Kingdom; 5 International Rescue Committee, Kampala, Uganda; 6 Mulago Palliative Care Unit, Mulago Hospital, Kampala, Uganda; 7 Uganda Cancer Society, Kampala, Uganda; 8 Yumbe Regional Referral Hospital, Yumbe, Uganda; 9 Dana-Farber Cancer Institute, Boston, MA, United States; 10 Palliative Care in Humanitarian Situations and Emergencies, Kampala, Uganda; 11 Stanford Medicine, Stanford, CA, United States; 12 Center for Health Services Research, Brandenburg Medical School Theodor Fontane, Neuruppin, Germany; 13 Imperial College London, London, United Kingdom

**Keywords:** digital health, palliative care, mobile health, humanitarian settings, refugee health

## Abstract

**Background:**

Palliative care is a key component of comprehensive humanitarian health; yet, access and service capacity remain limited in displacement settings, where fragile health systems struggle to meet the complex needs of people living with advanced illness. Digital health technologies have the potential to enhance the reach and delivery of palliative care; yet, their feasibility and acceptability in humanitarian settings remain underexplored.

**Objective:**

We evaluated the feasibility and acceptability of mPallCare, a mobile health intervention integrating patient-reported symptom and outcome monitoring with a clinician dashboard, to support palliative care delivery in the Bidibidi Refugee Settlement, Uganda.

**Methods:**

A 6-week, uncontrolled, exploratory concurrent mixed methods feasibility study was conducted, involving 32 participants with advanced cancer. Community health workers (ie, village health teams) used the mobile app to document patient-reported symptoms and multidimensional outcomes, which were accessible to clinical teams via a dashboard. Following the use of mPallCare, patient and clinical team participants participated in face-to-face interviews. Data collected via mPallCare were analyzed using descriptive statistics to assess feasibility (ie, compliance with reporting, with a feasibility threshold of ≥65% of scheduled reports), and interview data from a subsample of patient and clinical team participants were analyzed using framework analysis to assess acceptability.

**Results:**

Participants completed 84.9% (163/192) of symptom reports and 59.4% (266/448) of outcome reports, with a combined 67% (429/640) of all scheduled reports completed. A modest decline in engagement with report submissions occurred across the 6-week study period. Commonly reported symptoms included headache (27/32, 84.4%), muscle pain (27/32, 84.4%), and dizziness (26/32, 81.3%). Interview findings indicated strong acceptability among patients and clinicians, who described improved communication, enhanced symptom management, and greater continuity of care. Reported challenges included initial navigation difficulties, limited translation accuracy, and technical synchronization issues. Participants and clinical leaders identified the potential for integrating mPallCare within Uganda’s district health information system to strengthen data use and visibility of palliative care within health reporting structures.

**Conclusions:**

mPallCare is a feasible and acceptable digital health intervention for palliative care in a humanitarian setting. While initial uptake was high, sustaining engagement over time may require simplified reporting processes, enhanced language accessibility, and optimizing the mobile app’s connectivity and usability. This feasibility phase highlights key priorities for scale-up, including integration with existing health information systems and adaptation for sustained, equitable use across low-resource and displaced populations.

## Introduction

Globally, record numbers of people are living in displacement from natural and man-made disasters, leading to an increase in humanitarian and fragile settings [1]. Refugees’ and migrants’ experiences in their origin country, alongside their migration journey and subsequent entry to a host country, can influence physical and mental health needs [2]. Humanitarian settings are further associated with higher mortality and morbidity than the general population [1,3,4], alongside high levels of mental health conditions that can further negatively affect health outcomes [1]. These conditions, by the nature of their disease course, require long-term engagement through management, promotion, and prevention, alongside treatment, rehabilitation, and an integrated palliative care approach [5]. In practice, however, humanitarian agencies in emergencies often focus on “lifesaving” interventions, with little consideration for preventative, curative, or palliative measures for conditions not regarded as immediately life-threatening [6,7]. This apparent dichotomy in service provision means people living with advancing conditions endure suffering, with some referred to as the “expectant” (ie, expected to die, without any form of palliation or end-of-life care) [7,8]. This directly contravenes humanitarian principles, which explicitly require the prevention and alleviation of human suffering [9]. Moreover, displacement settings of all types have increasingly taken on some form of semipermanence, with marginal prospects for pathways to resolution, leaving millions of people spending many years to decades living in these conditions [10].

There is a crucial need to integrate palliative care into both emergency responses and as part of care delivery for people living in humanitarian and fragile settings [11]. Although the need for health services research in humanitarian settings remains significant [12], a paucity of data remains due to the unique and sometimes dangerous nature of humanitarian aid settings, a lack of familiarity by many academics, and the fact that research is often not a key priority for humanitarian organizations [1]. An emerging research area is the role of digital technologies, which are becoming a pivotal part of humanitarian responses, providing access to often limited and critical support in the context of crises. Digital tools are being increasingly used to address persistent challenges in the humanitarian sector, such as inclusion [13]. For palliative care in low-resource settings, digital technologies can provide a means of augmenting care and providing timely data to inform clinical and policymaker decision-making. To enhance care continuity, innovative digital health technologies can be leveraged in humanitarian crises and displaced populations [14] to support key tenets of palliative care delivery, including information sharing, decision-making, and communication [15]. Digital health approaches are common across palliative care providers [16], can improve service delivery around components such as pain management [17], and are acceptable across key stakeholders that include patients, caregivers, health and care professionals, and policymakers [18]. Digital health approaches, such as remote symptom monitoring, are also increasing across low-resource settings to support palliative care delivery [19].

This study arises from a research program focused on developing digital health for palliative care in sub-Saharan Africa. This study was initiated during the COVID-19 pandemic, when physical distancing was required to reduce transmission and digital approaches were encouraged in humanitarian settings [20]. A consortium of key care delivery actors in Uganda and the wider Africa region was involved, including the African Palliative Care Association (APCA), the United Nations Refugee Agency, the International Rescue Committee, and national and local care delivery organizations supporting palliative care. This study aimed to determine the feasibility and acceptability of a mobile phone app and clinician dashboard to support remote monitoring of the symptoms and outcomes of patients receiving palliative care. We present the findings of this novel digital health approach and lessons from its pilot testing to support palliative care delivery in the Bidibidi Refugee Settlement in Uganda.

## Methods

### Overview

**Figure 1.**
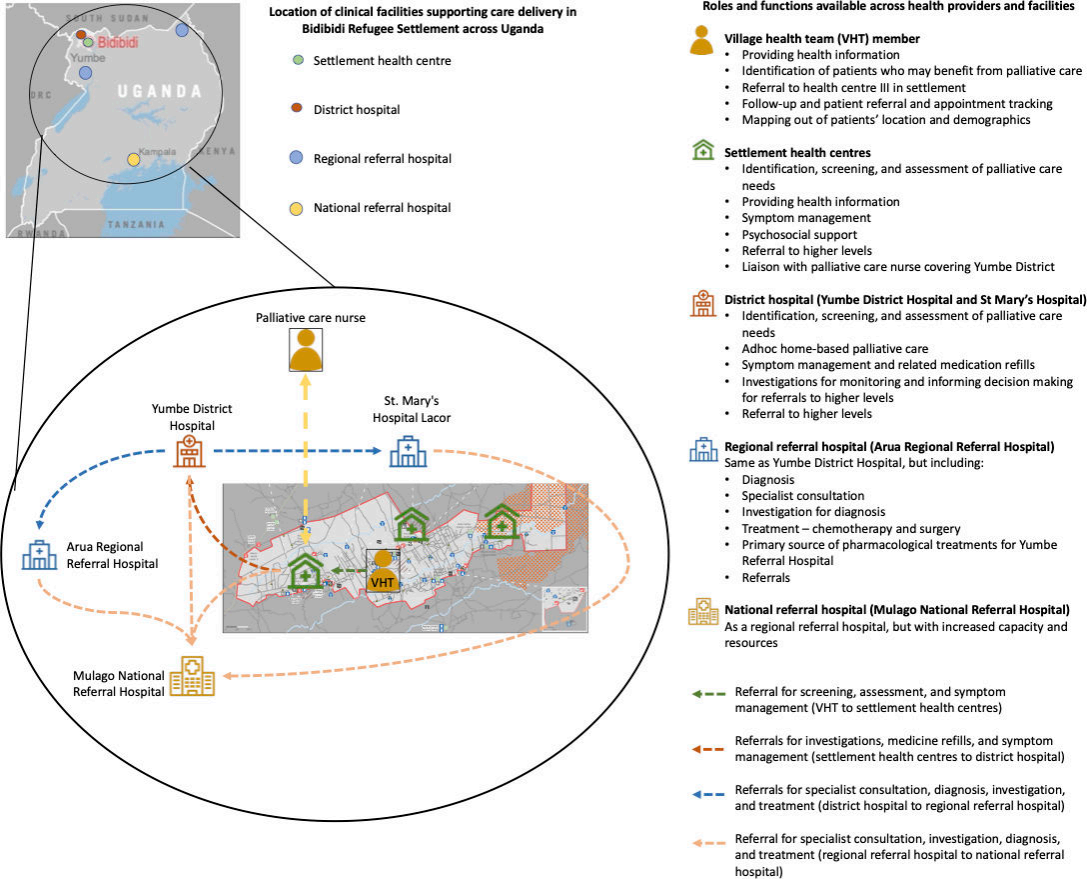
A schematic outlining the role of the village health team in identifying and supporting patients receiving palliative care in liaison with a palliative care nurse who supports referrals to a range of services available across various facilities, reflecting provision at the time of the study, with Yumbe District Hospital now a referral hospital with a hospital-based palliative care unit.

An uncontrolled, 6-week, exploratory concurrent mixed methods pilot feasibility and acceptability study was conducted, supplementing existing palliative care delivery in the Bidibidi Refugee Settlement. This design enabled complementary insights: quantitative findings identified patterns of engagement, while qualitative data explained underlying reasons and contextual influences. Uganda hosts one of the largest refugee populations globally, hosting more than 1.74 million refugees and asylum-seekers [21]. Most refugees come from South Sudan and the Democratic Republic of the Congo [21]. Bidibidi Refugee Settlement covers more than 250 km^2^ and is the largest settlement in the country, hosting more than 246,000 refugees across 5 areas, or zones, with a large majority of the population comprising women and children. Palliative care delivery within the settlement is currently overseen by one palliative care nurse based at a former district hospital (now regional referral hospital) in the nearest town, Yumbe (Figure 1). The palliative care nurse liaises with village health team (VHT) members and health care providers across the settlement. VHTs comprise community volunteers who help refugees and other community members access health services through, for example, providing health information and serving as a link between the village and health services. VHTs support palliative care delivery by identifying people with palliative care needs in the community and facilitating their access to care, in liaison with facility-based health workers in the settlement and the palliative care nurse. Opioid analgesics, including oral morphine, are available through the clinical teams within the settlement with palliative care nurse oversight. The structure of service delivery and referral relationships is summarized in Figure 1.

### Intervention Development

The study is positioned within the first 2 stages of the Medical Research Council Framework for the Development and Testing of Complex Interventions [22], that is, to develop a novel intervention and to determine its feasibility and potential effectiveness in humanitarian settings. The project team included applied health researchers (University of Leeds, Makerere University, and Palliative Care Education and Research Consortium), APCA, palliative care providers (New Life Hospice, Yumbe Regional Referral Hospital Palliative Care Unit, and National South Sudan Palliative Care Association), a patient advocacy organization (the Uganda Cancer Society), and a digital technology company (Universal Doctors).

**Figure 2.**
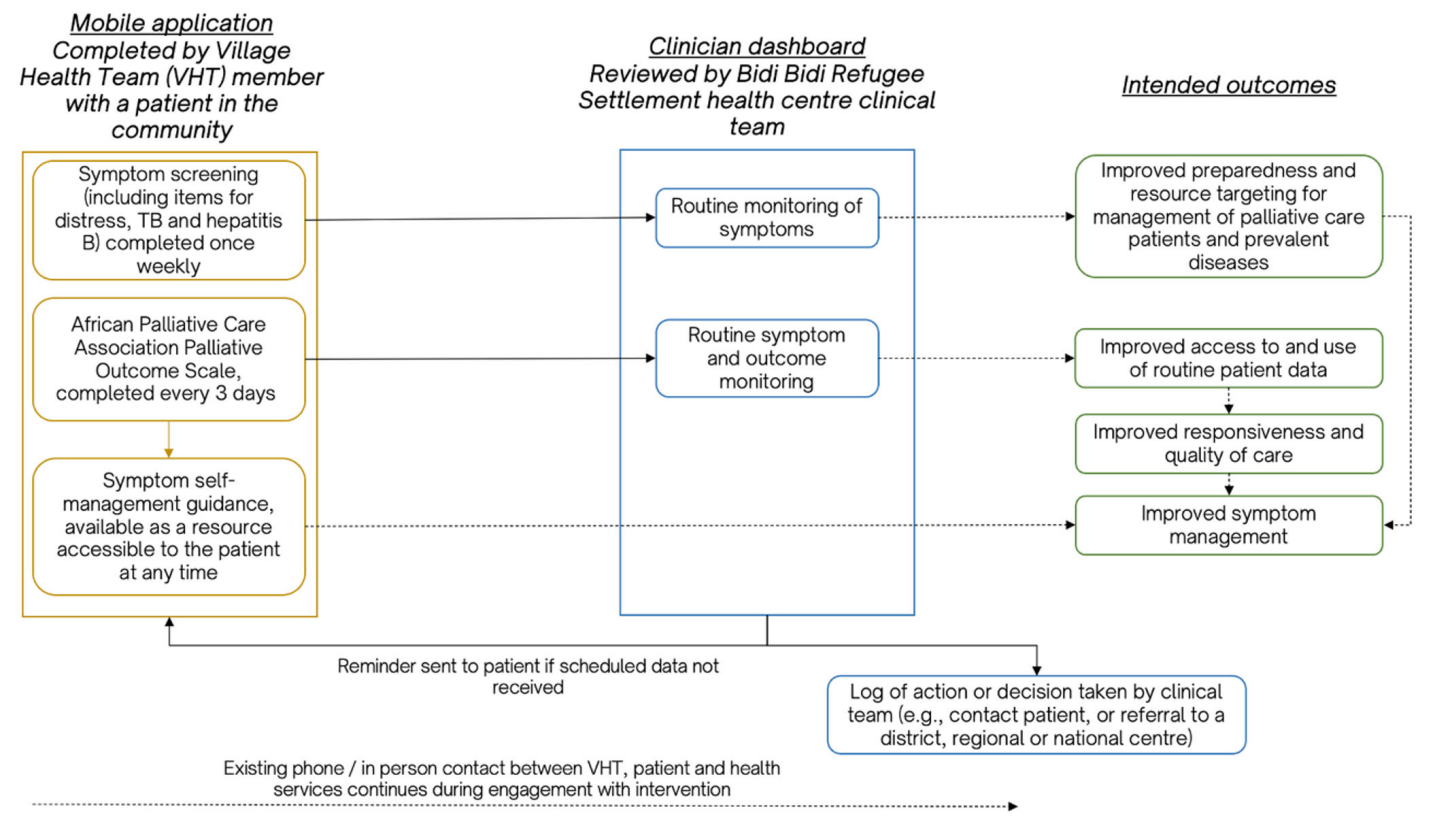
Schematic to show the structure of the mobile phone app and dashboard discussed with key stakeholders, alongside preliminary hypothesized and intended outcomes.

An initial platform structure using a mobile phone app and clinician dashboard was developed following guidelines for digital health use in palliative care in Africa previously developed by the team (Figure 2) [18]. This included the need to support reporting on physical, psychological, social, spiritual and practical symptoms, the provision of information about living with illness and enabling communication with palliative care services [18]. The resulting platform gathers patient-level symptom and outcome data via the mobile phone app, in addition to providing access to self-management guidance and support (ie, guidance on the management of common symptoms using both video- and text-based content). This approach is underpinned by evidence highlighting that routine symptom and outcome monitoring and the provision of self-management resources can improve symptom management and quality of care delivery for people with palliative care needs [23,24].

To refine and finalize the digital health approach, an advisory group was formed and consulted. The group was facilitated by the APCA and included clinic staff from New Life Hospice and Yumbe Regional Referral Hospital Palliative Care Unit, Ministry of Health representatives, global health security and infectious disease experts from the Infectious Diseases Institute in Uganda, patient representatives supported by the Uganda Cancer Society, and gender, diversity, and inclusion experts. During a stakeholders workshop in Yumbe District, Uganda, and hosted by the APCA, the following topics were discussed: alignment of the intervention and its content with the refugee population, review and development of symptom and outcome measures that could be collected via the mobile phone app, and developing an implementation plan that built on and strengthened existing activity, while ensuring alignment with the Uganda Health Sector Integrated Refugee Response Plan [25]. The Refugee Response Plan recognizes the need for parallel provision of services across refugee and host communities, highlighting health technologies and health information as key pillars in developing health care for refugee populations. Following the meeting, the intervention was refined, with the symptom checklist expanded to ensure the inclusion of symptoms indicative of tuberculosis and hepatitis B, both prevalent in the settlement. Furthermore, options for the inclusion of symptom and outcome scales were discussed, including which items would be included in the mobile phone app, balancing established protocols for using the tools and existing workflows of VHTs in the community setting.

### The mPallCare Intervention

mPallCare comprises 2 components: a mobile phone app to gather patient-reported data that is completed by a VHT member (ie, a volunteer community health worker) from the settlement clinical team, and a web-based clinical dashboard that displays the data gathered from the mobile app for clinical teams (as shown in Figure 2). Together, using the mobile app, a VHT and patient complete 2 forms: a symptom screening questionnaire and a multidimensional outcome measure designed for palliative care in Africa [26]. The symptom screening questionnaire was developed during the advisory group workshop and included symptoms commonly reported by patients receiving palliative care (eg, pain, dyspnea, nausea, and vomiting). Items also included symptoms indicative of COVID-19, 4 symptom screening items for tuberculosis as recommended by the World Health Organization [27], and 2 items related to symptoms that could be suggestive of hepatitis B (ie, clay-colored stools and yellowing of the skin; refer to Multimedia Appendix 1 for symptom screening items). Respondents were asked to respond (yes or no) to a total of 17 items, reporting whether they had been present over the previous week. The multidimensional outcome scale used is the APCA Palliative Outcome Scale (POS) [26], which assesses key palliative care domains using numeric rating scales. It includes 7 patient-focused questions that include the following:

1. Pain: captured using a scale (0=not at all; 5=overwhelmingly)
2. Other symptoms: “Have you been affected by other symptoms (such as nausea, breathlessness, etc)?" (0=not at all; 5=overwhelmingly)
3. Concern: Have you been feeling worried about your illness in the past 3 days? (0=not at all; 5=overwhelming worry)
4. Feelings: Over the past 3 days, have you been able to share how you are feeling with your family or friends? (0=not at all; 5=yes, I’ve talked freely)
5. Life: Over the past 3 days, have you felt that life was worthwhile? (0=no, not at all; 5=yes, all the time)
6. Peace: Over the past 3 days, have you felt at peace? (0=no, not at all; 5=yes, all the time)
7. Advice: Have you had enough help and advice for your family to plan for the future? (0=not at all; 5=as much as wanted)

Data reported via the mobile app are then displayed on a clinical dashboard, enabling a settlement clinical team to monitor patient symptoms and outcomes routinely, determine any changes or problematic symptoms, and devise an appropriate response plan. This may involve coordinating with external palliative care specialists located beyond the settlements to ensure comprehensive cancer care. The symptom and outcome reports were available in the mobile phone app in 4 languages (English, French, Swahili, and Juba Arabic), developed through forward and backward translation by professional translation services in Uganda. Aligned with Figure 2, this approach seeks to support the timely identification and management of symptoms and issues, as well as inform the coordination of, and referrals to, additional services and clinical sites when needed. This may potentially influence reductions in any symptom burden experienced by patients, as well as increase coordination and care continuity.

mPallCare also includes self-management resources developed by the research team, including an expert in physical activity in advanced disease (SB). Text- and graphic-based self-management content was developed for pain (eg, managing medications, relaxation, and distraction; adapted for the context from earlier work by the team) [28], breathlessness, fatigue and energy, and mobility. Following the submission of symptom and outcome reports using the mobile phone app, self-management related to any reported symptoms is displayed (eg, reporting pain displays pain self-management information). However, patients and health workers could access self-management information for all symptoms at any time as a standalone resource within the mobile phone app. Once reports are submitted, the data are available immediately via the clinic dashboard. The dashboard presents individual lines for each patient report received, with a flag system (ie, a red flag placed next to a patient report on the main dashboard) where any symptom or outcome item has been reported. Any member of the clinical team can review a report submitted by a VHT. On reviewing a report, the clinical team member is asked to detail what action they will take (ie, contact the patient, speak to a colleague, or do nothing), and there is space to add additional notes. This feature was included to enable other clinic team members to understand actions in response to reviewing a report.

A strong focus was placed on the user-friendliness of the graphic interface on the mobile phone app by the software developers. The technical requirements of the mobile phone app were devised around the setting in which its use was intended and made available for an Android smartphone with Android OS 4.0.3 or above. A secure server was set up to receive data reported via the mobile phone app. A web-based backend was developed to allow the clinical team members to log in and view the data submitted. All patient data submitted was deidentified before analysis to protect privacy and confidentiality.

### Study Design

The mixed methods study sought to determine the feasibility of collecting palliative care patient-reported outcome data and symptomatology mapping, alongside qualitative investigation of the acceptability of the intervention to target users.

### Sample

Participants were selected using a purposive convenience sampling approach, with all eligible patients on existing palliative care lists invited consecutively until the target sample was reached. The most proximate entry point of care for patients receiving palliative care in Bidibidi Refugee Settlement was from the camp-based health facilities via the Arua Regional Referral Hospital. From the hospital, patients are typically referred to New Life Hospice (based in Arua, Uganda) or Yumbe Regional Referral Hospital palliative care unit. We noted that it was common for New Life Hospice to screen camp-based patients from Bidibidi Refugee Settlement who were then referred to Yumbe Regional Referral Hospital palliative care unit, which is closer to their residence. Eligible participants were identified collaboratively with the incumbent health service provider (International Rescue Committee) from existing patient lists of those receiving care, generating an initial list of 32 eligible camp-based patients on the palliative care program from the 2 services, including both refugees and displaced Ugandan nationals. Both refugees and Ugandan nationals were included to reflect the integrated service delivery model operating across settlement and host communities, consistent with Uganda’s national policy promoting equitable access to health services. This approach aligns with broader strategic frameworks developed by the United Na Country Team in Uganda and the Ministry of Health to strengthen integration between host and refugee populations and promote peaceful coexistence. This list was continually updated during the recruitment period to account for attrition through patient deaths or relocation from the settlement. The eligibility criteria used to generate the list are outlined in Textbox 1. The final sample of 32 participants reflects all patient participants approached who met the criteria and consented during the recruitment period.

Textbox 1. **Eligibility criteria for patient study participants.**

**Inclusion criteria**

- Aged 18 years or older
- South Sudanese or Ugandan nationals
- Receiving hospice care or palliative care from Arua Regional Referral Hospital, Yumbe District Hospital, or New Life Hospice
- Living with advanced cancer, defined as those with metastatic cancer (where, if possible, determined through histological, cytological, or radiological evidence) and/or receiving anticancer therapy with palliative intent
- Estimation of expected prognosis between 3 and 12 months
- Able to read and understand one of the languages available on the mobile phone app (ie, English, French, Swahili, or Juba Arabic) or have a carer who can read and understand one of the languages available
- Able to provide informed consent to participate and have sufficient cognitive ability to provide informed consent

**Exclusion criteria**

- Patients who are too ill to endure study procedures
- Dyads with visual impairments who are unable to read screen-based information
- Patients who may wish to return to their home countries over the next 3 months

### Procedure

Before the commencement of patient enrollment, a training session was delivered for VHTs in May 2022. VHTs were provided with training manuals developed to support navigation and use of the mobile phone app, and an in-person training session took place. The training was attended by representatives from the regional referral hospital, the district health office, the Ministry of Health of Uganda and the Office of the United Nations High Commissioner for Refugees to ensure awareness of the project and commitment to its delivery. The care team for the settlement approached potential participants and generated a list of people who agreed to participate in the study. VHTs supporting participants across the different zones in the settlement attended the training session ahead of the study commencement. In total, 20 VHTs were trained on the study objectives, ethical principles (confidentiality and informed consent), procedures for administering symptom and outcome tools, and step-by-step use of the mPallCare app (research team of the University of Leeds, African Palliative Care Association, and UniversalDoctor) and dashboard. An IT specialist from the APCA led practical sessions that included simulated data entry (ie, using the app for submitting the patient outcome and symptom reports), troubleshooting exercises, and accessing self-management symptom guidance. From the training, it was highlighted that there was a risk that devices provided for the study could be at risk of being stolen, so they were retained in health centers rather than VHT homes when they were not being used. VHTs also requested a paper copy of the self-management resources, which were provided for the study. During the training, the initial list of identified patients was discussed and confirmed in partnership with representatives from Yumbe District Hospital, the International Rescue Committee, Arua Regional Referral Hospital, and New Life Hospice. Initial testing focused on advanced cancer, a common disease group in populations in refugee settlements in Uganda, alongside chronic hepatitis B, diabetes, severe injuries, and sickle cell anemia [29].

Following training, VHTs supported the identification and recruitment of eligible patients. Active recruitment and enrollment of patients took place between August and October 2022. VHTs worked with clinical teams to review health facility patient lists to identify those who met the eligibility criteria. Where eligible patients were identified, a VHT discussed the study, what participation entailed, potential risks and benefits of participation, and the process of data flow from the mobile phone app to the clinic, including where data are stored. If the patient agreed to participate, consent was obtained with a signature or thumbprint. We sought to recruit between 30 and 35 patients receiving palliative care, aligned with sample size requirements for feasibility studies [30]. For those who enrolled in the study, a baseline demographic questionnaire was completed capturing a participant’s age, gender, highest level of education, home location (zone and block or TIN number), primary diagnosis, family size, Demographic and Health Surveys measure of socioeconomic status [31], and whether the patient had access to a mobile phone (as a proxy for familiarity with digital devices and to determine the future feasibility of patient-reported data). The Eastern Cooperative Oncology Group Scale of Performance Status [32] is also captured at this point, which describes a person’s functional status based on their capacity for self-care, engagement in daily activities, and physical capabilities such as walking and working. A grade is provided from 0 (fully active, able to carry on all predisease performance without restriction) to 5 (dead), with graduated levels of function between the two values. Responses to the baseline demographic questionnaire were recorded by VHTs using a purpose-designed Microsoft Excel spreadsheet with logic and consistency checks to reduce errors.

Following the capture of sociodemographic information, the VHT worked through both the outcome and symptom reports with a patient. Following initial completion, it was expected that a VHT would complete 1 symptom report per week for each patient over the 6-week study period (6 reports×32 participants=192 expected symptom reports) and 1 outcome report every 3 days (approximately 14 reports×32 participants=448 expected outcome reports). In total, 640 reports were planned across the study period. VHTs visited patients every 3 days to complete and update the outcome report, and once weekly to complete the symptom screening report for up to 6 weeks. During the study period, an IT specialist from the APCA monitored registrations of new patient participants to ensure active recruitment and responded to any queries related to device functionality and data synchronization.

Following the completion of 6 weeks of reporting, clinical officers from Bidibidi Refugee Settlement who were members of the research team (RR, NM, or TM; all male) conducted semistructured face-to-face interviews with patient participants at their homes. The semistructured format allowed for consistent coverage of key areas—such as patient participants’ experience of completing reports with VHTs, exploring the perception of how data would be used, and any perceived influence it had on the care they received. Interviewers remained flexible around the presence of family members and caregivers during interviews. An interpretivist paradigm was deemed the most suitable approach to the qualitative component of the study, due to the nature of the exploration of feasibility and acceptability and the necessity of understanding individual experiences of mPallCare from the viewpoint of those who interacted and used the platform. This perspective emphasizes the significance of subjective interpretations, personal perceptions, and the meanings individuals attribute to their experiences [33].

A purposive sampling strategy was used to ensure representation of patient participants with diverse demographic and clinical characteristics relevant to the study aims, drawn purposively from the same feasibility cohort. We sought to capture variation across age and diagnoses (ie, different cancer types) in a subsample of 10 patients. We sought to understand their experience of the mobile phone app being used to obtain symptom and outcome data. Alongside patient interviews, clinical staff and VHTs involved at each site (n=10) were purposively sampled by site (geographic zones within the Bidibidi Refugee Settlement) and role (ie, VHT, clinical officer, and nurse) and participated in face-to-face interviews to explore their views of how the app had supported patient care and the experience of its implementation in routine care. Our sampling approach did not seek to achieve data saturation but rather capture a breadth of perspectives that could provide practical insights into user experience and inform future implementation and scale-up. This approach focused on identifying variation and key issues influencing acceptability and feasibility. Interviews were conducted by a female member of the research team with specialist palliative care clinical expertise and undertaking an MSc in Palliative Medicine (LN). Patient and clinical staff participants were not known to the research team. The research team members were fluent in the languages used during interviews. All interviews were audio-recorded and transcribed verbatim in the language used, then translated into English where required by the researcher who conducted the interview and included in parentheses alongside the original transcription. Transcripts were then checked against audio files by a second member of the research team involved in conducting interviews to ensure accuracy in the translation and transcription.

### Analysis

All statistical analyses were conducted in SPSS (version 28.0; IBM Corp). Descriptive statistics were run on the demographic and clinical characteristics of the individuals who completed outcome and symptom reports. Compliance with the outcome and symptom reporting protocol was reported in terms of completed reports. Graphical representations were developed to chart app usage over the 6 weeks. Individual mean scores were calculated, as well as a grand mean for each outcome variable. The change in the outcome measures of Concern, Feelings, Life, Peace, and Advice was charted over time. Feasibility is defined as participants completing ≥65% of scheduled reports, reflecting a pragmatic, context-appropriate target informed by prior studies reporting completion rates between 60% and 80% in studies adopting digital health approaches in the collection of patient-reported outcomes for palliative care populations: for example, completion rates of 58% for weekly patient-reported online questionnaires [34], 73% for mobile phone–based data capture [35], and 78.4% of outcomes data via a patient portal [36]. While there are no comparable studies in the context of humanitarian settings, the conservative threshold reflects broader digital health implementation challenges common in the context of humanitarian settings [37], including availability, access, affordability, and rights. Acceptability of the intervention was explored through both patient and clinical team interviews on the experience of using the mPallCare over the study period.

Interview transcripts from patient and clinical staff were imported into NVivo 12 (QSR International) for framework analysis [38]. This approach guided a structured, transparent, and systematic comparison across participant groups while remaining grounded in participants’ accounts. The framework approach also enabled the organization of data around predefined domains of interest (ie, usability, communication, and integration with care) while permitting the inductive identification of emergent themes reflecting user experience and contextual factors. MA and AA analyzed the interview transcripts. Separate analyses were undertaken for transcripts derived from patient interviews and those completed with clinical team staff and VHTs. For both sets of data, the same approach was adopted. A coding frame was developed using both deductive and inductive approaches, with an initial framework developed from a selection of 4 transcripts. For both, these were selected to ensure variation in the clinical sites and participants (for patients, varying by sex and age, and for clinical team members by role). The developed frameworks were discussed and, where necessary, adapted by the wider team, after which the remainder of the transcripts were analyzed. Where differences or uncertainty arose, these were discussed with the wider team. Comparative analysis in the patient and clinical team frameworks enabled common themes to be identified, as well as sample divergences. Illustrative quotes are reported for each theme, alongside the study participant ID and site, to demonstrate reporting from across the sample breadth.

### Ethical Considerations

The protocol, interview guide, and consent materials were reviewed and approved by the Hospice Africa Uganda Research Ethics Committee (reference HAU-2021‐4) and complied with the principles outlined in the Declaration of Helsinki. All study participants received verbal and written information about the study objectives, procedures, potential risks, and their right to withdraw at any time without consequence to their care. For participants with limited literacy, consent was read aloud in their preferred language and consent was recorded through signature or thumbprint. All data were deidentified prior to analysis, stored on encrypted servers, and accessible only to the research team. No financial compensation was provided to participants. The paper has been compiled in accordance with the COREQ (Consolidated Criteria for Reporting Qualitative Research; Checklist 1), ensuring transparency and completeness in the reporting of study design, conduct, and analysis.

## Results

### Overview

In total, 32 participants with advanced cancer took part, with the majority of these being female (n=19, 59.4%), Christian (n=19, 59.4%), and married (n=17, 53.1%). The ages of the participants ranged from 18 to 77 years, with an average of 48.5 (SD 16.9) years. The primary caregiver was most often their spouse (n=16, 50%), but children and parents were also commonly reported. Most participants also did not have access to a mobile phone (n=18, 56.3%). An initial list of participants comprising camp-based patients on the palliative care program included additional participants to accommodate challenges in recruitment. Participants initially identified on the list included those who died (n=6) and those who left the settlement (n=4) before study commencement. A breakdown of the demographic and clinical characteristics of the participants can be found in Table 1.

**Table 1.** Demographic and clinical characteristics (N=32).

Variables	Values
Age (y), mean (SD)	48.5 (16.9)
ECOG^a^ functional performance, mean (SD)	1.6 (0.8)
Family size, mean (SD)	9.2 (2.9)
Nationality, n (%)	
South Sudanese	13 (40.6)
Ugandan	19 (59.4)
Sex, n (%)	
Male	13 (40.6)
Female	19 (59.4)
Religion, n (%)	
Christian	19 (59.4)
Muslim	13 (40.6)
Marital status, n (%)	
Married	17 (53.1)
Single, divorced or separated, or widowed	15 (46.9)
Highest level of education, n (%)	
None	22 (68.8)
Primary or secondary	10 (31.3)
Caregiver relationship, n (%)	
Spouse	16 (50.0)
Child	10 (31.3)
Parent	6 (18.8)
Access to mobile phone, n (%)	
No	18 (56.3)
Yes	14 (43.8)

a ECOG: Eastern Cooperative Oncology Group.

### Outcome and Symptom Report Data

All patient participants (N=32) completed symptom and outcome reports. From a total of 192 possible symptom reports (32 participants × 6 time points), participants completed 163 reports (84.9%), an average of 5.1 reports per participant, with a range of 2 to 11 reports. From a total of 448 possible outcome reports (32 participants × 14 time points), participants completed 266 reports (59.4%), an average of 8.3 reports per person, with a range of 3 to 13 reports. In total, a combined 67% (429/640) of all scheduled reports were completed.

**Figure 3.**
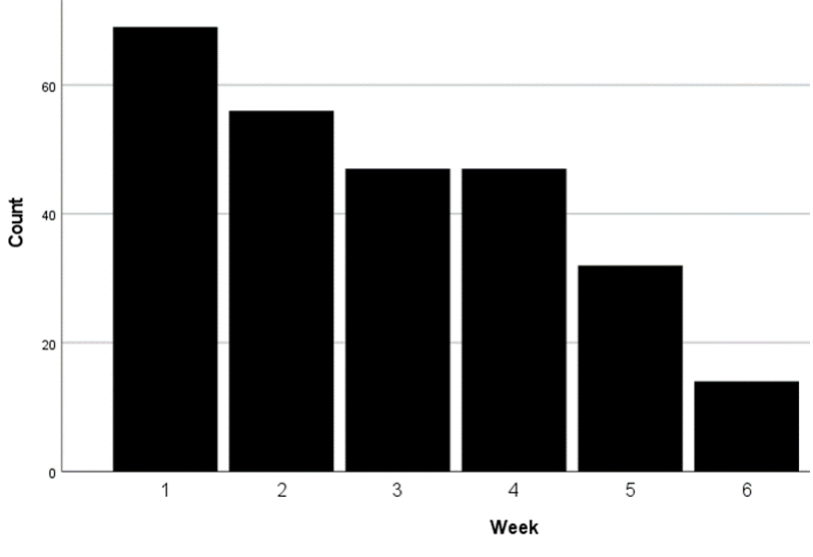
Frequency of responses submitted via the patient mobile phone app across the 6-week study period.

Figure 3 presents the frequency of responses from participants over the course of the study, which shows that as the study progressed, responses declined. Detailed findings from patient and clinical team interviews are provided below. Decline in engagement was reported relating to practical and technical barriers. Early difficulties with navigation and translation occasionally delayed data entry, and intermittent connectivity and dashboard errors resulted in missing or repeated reports. VHTs also described periods when device availability or competing health outreach duties limited the frequency of visits to patients. Collectively, operational factors may have contributed to the reduced number of submissions observed toward the end of the study.

**Figure 4.**
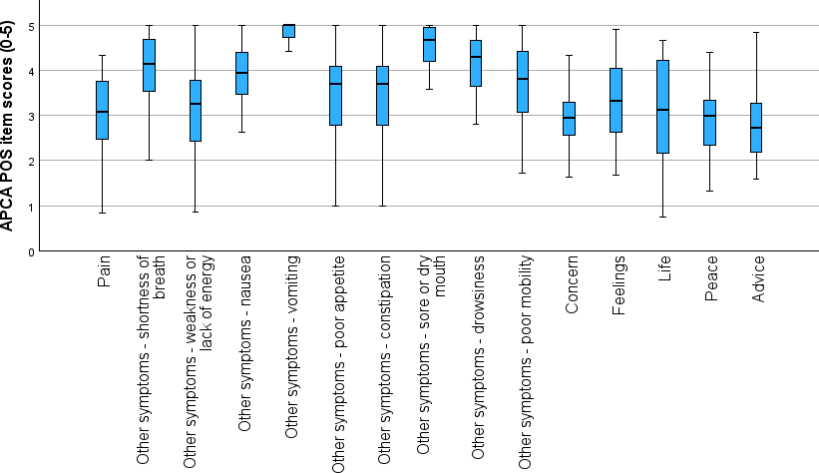
Boxplots showing the mean and range of scores of participants across the outcome measures, which include pain, other symptoms (shortness of breath, weakness or lack of energy, nausea, vomiting, poor appetite, constipation, sore or dry mouth, drowsiness, and poor mobility), Concern, Feelings, Life, Peace, and Advice. Higher scores indicate a more positive outcome and lower scores a more negative outcome. APCA: African Palliative Care Association; POS: Palliative Outcome Scale.

**Figure 5.**
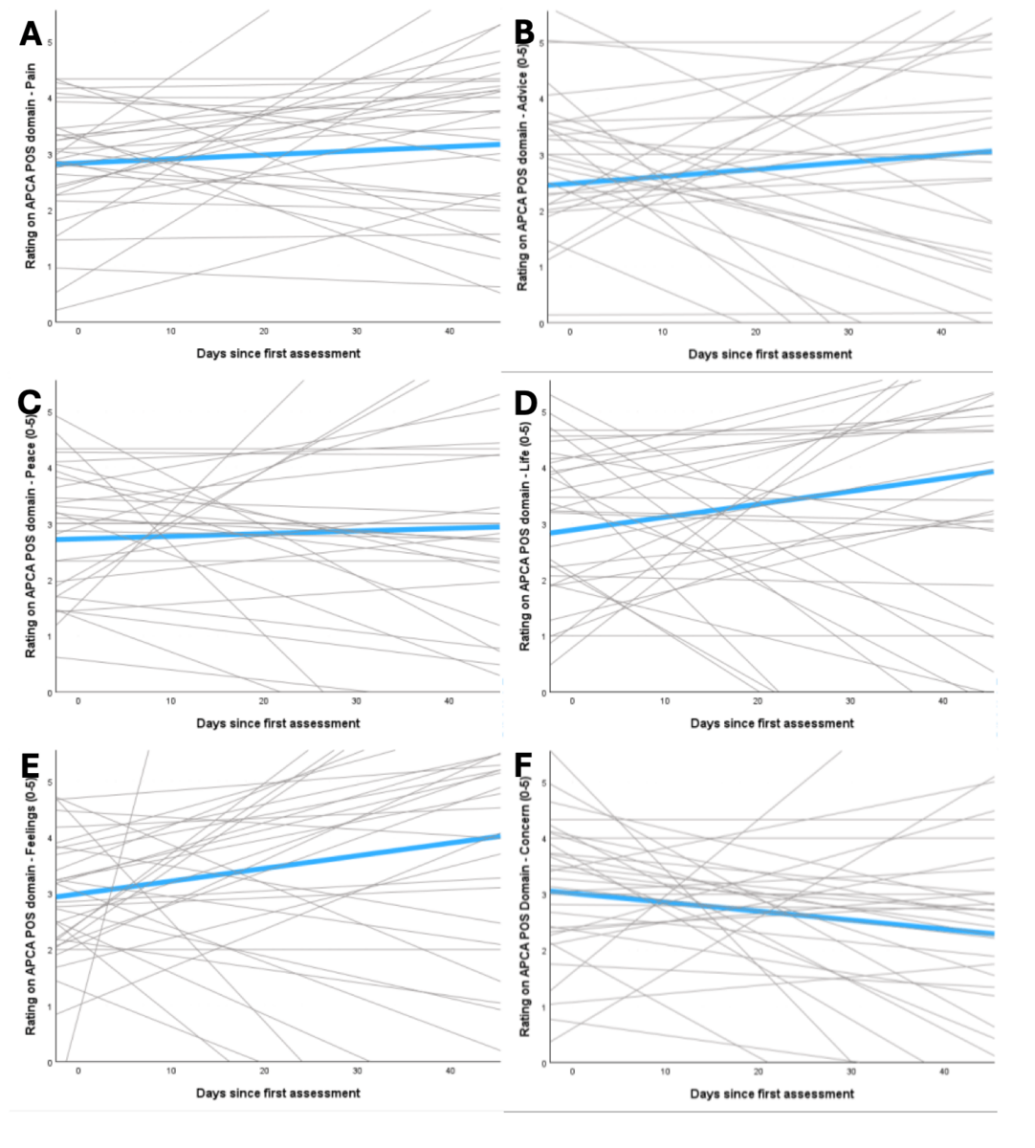
Changes in participants’ self-reported scores on the African Palliative Care Association Palliative Outcome Scale domains over the study period, with each gray line representing an individual participant’s fitted trajectory of change in scores over time (in days). The blue line represents the overall fitted trajectory for the sample. Higher scores indicate a more positive outcome in each domain (A=Pain, B=Concern, C=Feelings, D=Life, E=Peace, F=Advice). APCA: African Palliative Care Association; POS: Palliative Outcome Scale.

Figure 4 presents the individual mean scores of each outcome measure across the study period. The symptoms of vomiting, sore or dry mouth, and drowsiness had the highest scores, whereas the symptoms of weakness or lack of energy, constipation, and poor appetite had the lowest symptom scores, although these also had a wider range of scores. The scores for the measures for Feelings, Concern, Life, Peace, and Advice were clustered around 3.0. However, Figure 5 suggests that each of the outcomes of Pain, Feelings, Life, Peace, and Advice improves throughout the study but that Concerns around their illness increased over the study. However, these graphs also indicate there is substantial variability in individual scores, with some individual scores decreasing or increasing over time.

Table 2 outlines the symptoms reported by participants over the course of the study. Across all participants, the most commonly reported symptoms were headache, muscle pain, and dizziness; the least reported were colored stools, yellowing skin, and diarrhea, with fatigue not reported at all. The most reported symptoms over the course of the study were headaches, chest pain, weight loss, and abdominal pain, with diarrhea and foot blisters being the least commonly reported.

**Table 2.** Symptoms reported by participants.

Symptom	Number of participants reporting symptom (%)	Number of times symptom reported	Of those reporting symptom, average number reported
Headache	27 (84.4)	90	3.3
Muscle pain	27 (84.4)	73	2.7
Dizziness	26 (81.3)	76	2.9
Chills	25 (78.1)	59	2.4
Weight loss	24 (75.0)	75	3.1
Chest pain	24 (75.0)	75	3.1
Loss of appetite	24 (75.0)	69	2.9
Night sweats	22 (68.8)	35	1.6
Abdominal pain	22 (68.8)	68	3.1
Fever	20 (62.5)	40	2.0
Shortness of breath	19 (59.4)	46	2.4
Confusion	18 (56.3)	46	2.6
Cough	17 (53.1)	38	2.2
Eye soreness	14 (43.8)	32	2.3
Loss of smell	13 (40.6)	25	1.9
Hoarse voice	13 (40.6)	24	1.9
Painful throat	10 (31.3)	17	1.7
Nausea and vomiting	9 (28.1)	16	1.8
Welts or swelling	9 (28.1)	16	1.8
Prolonged cough	6 (18.8)	13	2.2
Feet blisters	6 (18.8)	8	1.3
Diarrhea	3 (9.4)	4	1.3
Colored stools	2 (6.3)	3	1.5
Yellowing skin	2 (6.3)	5	2.5
Fatigue	0 (0.0%)	--	--

### Interviews With Patients and Clinical Teams

In total, 20 qualitative interviews were conducted; 10 with patient participants and 10 with members of the clinical team, including VHT members, nurses, and clinical officers. From the patient interviews, three key themes were generated from the analysis: (1) ease of use and perception of app usefulness, (2) understanding of the questions and perception of data use, and (3) impact of the intervention on health and care. From health professional interviews, three themes were generated from the analysis, namely (1) perception of impact of the intervention on patient care, (2) experiences of implementation, and (3) data robustness and reporting. Representative quotations are presented within the Results to illustrate key findings within each theme. The duration of interviews ranged from 20 minutes to 1 hour and 33 minutes for patient participants and 18 to 44 minutes for clinical team interviews. Exemplar quotes from patient and clinical team interviews are presented in Table 3.

**Table 3.** Exemplar quotes to accompany the narrative of findings from patient and clinical team interviews.

Theme	Supporting quotes
Patient interview findings	
Ease of use and perception of app usefulness	Quote 1: “The fact that it was my first encounter with a smartphone, I found it challenging in the beginning, however with time with the help of the VHT I was able to somehow learn how to use the phone.” (Patient participant 4, female 77, cervical cancer) Quote 2: “...to be able to communicate better to a wider population especially those who don’t understand English, picture illustrations would be added to make people understand better.” (Patient participant 2, female 40, cancer of the eye) Quote 3: “I found some difficulties, especially with some of the words which I couldn’t as well understand. However, with the help of the VHT, they were interpreted. My suggestion is that the app should be changed to a language that we usually use here, for example, Juba Arabic and Bari to make it simpler.” (Patient participant 2, female 40, cancer of the eye)
Understanding of the questions and perception of data use	Quote 4: “...some other things were okay to use that method and others were hard, for example, say, if say I’m worried, : “I found some difficulties, especially with some of the words which I couldn’t as well understand. However, with the help of the VHT, they were interpreted. My suggestion is that the app should be changed to a language that we usually use here, for example, Juba Arabic and Bari to make it simpler.” (Patient participant 2, female 40, cancer of the eye) Quote 5: “...On about two occasions I received a call from the doctors saying that I needed to be reviewed, this made me think that they’d based on what I’d sent them to make that decision, hence, according to me, the scores when seen by the doctors were used make decisions on how I felt. I was told that they looked at the scores and indeed they had meaning, green, yellow and red; all had different meanings. The scores that I had sent at that time were yellow, I was told yellow was a sign that my sickness was increasing and so, there was need to review me so that I don’t enter red or get worse.” (Patient participant 2, female 40, cancer of the eye)
Impact of the intervention on health and care	Quote 6: “...I began becoming open to the doctors and felt it easy to tell me my plight as I felt it, the fear of the doctors went away while using the app, I could say that the communication gap really went away during this time.” (Patient participant 6, male 40, colorectal cancer) Quote 7: “For sure, during that period, the VHT used to visit me frequently, sometimes 3 or 4 times to check on me, that made me feel closer to him and know him more. During those times, drugs used to come on time, and it became hard to miss as the doctors were always in close contact with me especially when I had not been able to reach the health center. Even the times when the doctor calls you to the health center, I used not to delay he knows how I feel so I just go come back in no time.” (Patient participant 4, female 77, cervical cancer) Quote 8: “It has created a strong bond between me and my doctors. For example, I am better understood in the health facility because I’m constantly monitored by the doctors. This as well led to me not delaying in the facility because the doctors already know how I feel even before I get there, this saves me the trouble of explaining myself every time when I come to the facility.” (Patient Participant 10, female 40, breast cancer) Quote 9: “There’s that part that talked about how to manage a difficulty in breathing especially when working. When I followed the advice, the app gave me on how to control my breathing, that really improved my symptoms, and I was able to even become more productive. The symptom management further helped me realize that I can live with the disease which initially I thought it was a death sentence to me.” (Patient participant 10, female 40, breast cancer) Quote 10: “There was a question that accessed my mental health status, especially how I related with family members, if I talk to them and how close I was, this reminded me to always talk to them in case I’m not feeling okay or worried.” (Patient Participant 2, female 40, cancer of the eye)
Clinical team interview findings	
Perception of impact of the intervention on patient care	Quote 11: “I think I mentioned before we were able to stay in touch with patients even before their appointments; before we would only interact with these patients on their appointments right now they are being seen twice so the frequency with which you’re interacting with the patient and progress is more by about 4 times instead of a one month review, you can review your patient like 4 times a month; a patient is interacting with you more not only on their treatments and what but also on other issues like psycho-socio, you’re visiting them so definitely the connection was increased.” (Health team participant 2, clinical officer) Quote 12: “...it changed somehow because it was, I was actually now dealing more with, relying on what the VHT tells me and you know sometimes this information as you were asking whether they benefitted the patients to some extent you would wish this patient talks directly and you listen from this patient so that you get the quality of the information.” (Health team participant 3, palliative care nurse)
Experiences of implementation	Quote 13: “The design of the app was good but the challenge I found there was the severity of the sickness, like slightly, moderate severe, more severe those things in the beginning, translating it in the local language was a challenge. But now I myself I started making research from my colleagues; what do you mean by moderate in our language, severe and slightly and after coming up with the meaning of those word in our local language, I would then translate to the patients, then it became familiar but, in the beginning, these were a bit challenging.” (Health team participant 8, village health team member) Quote 14: “That challenge came with one of the patients; the patient was started on the program from the dashboard he just disappeared from the app, from the VHT side the information is there on the app again they told me that was a technical problem otherwise that thing of VHT not submitting a report; No. they have submitted the reports.” (Health team participant 3, palliative care nurse)
Data robustness and reporting	Quote 15: “Then the option, on the option of the actions to do for the patient depending on the assessment, if we could broaden it a bit. We had where either you go speak to the client as a professional, then you are going to call and then others; so, in the others is where you would put referrals. I think if we could have like referrals captured separately or linkage captured separately so that patients are linked to other sources of service like psycho-socio support; others were linked to protection; others were linked to livelihood so if we could have where we can capture this then it would be great for the design.” (Health team participant 8, village health team member) Quote 16: “...if we can along the way if you can find a way of having it reflect some elements of the DHIS too in the information system so that we can actually have this data fed into our reporting into the ministry as well. I know that will take improvements on both sides; an improvement onto updating and making our data collection for palliative care in the Ministry of Health system also up to date because right now it also looks shallow; it is also shallow so maybe that will take two sides so that when you have this patient’s data you can easily extract it to inform the district and the Ministry of Health in terms of programs.” (Health team participant 2, clinical officer)

### Patient Interview Findings

#### Theme 1: Ease of Use and Perception of App Usefulness

The mobile phone app was accessed with the help of the VHT, in keeping with the intended use. Initial difficulties in navigating the app and understanding the input of data were easily overcome with support from a VHT and with repeated use (Quote 1 in Table 3). Some participants reported that self-management resources could be enhanced through the inclusion of visual aids such as pictures, as well as improving the format and presentation of information (ie, increasing font size and wider availability of accessibility options, such as pictorial and audio formats; Quote 2 in Table 3). All participants found the mobile phone app useful and particularly appreciated that symptom and outcome items were easy to understand, requiring only minimal explanations from the VHT, and relevant to their condition (Quote 3 in Table 3). Some participants indicated that translation into additional local languages may also improve the app.

#### Theme 2: Understanding of the Questions and Perception of Data Use

Participants generally agreed that the questions were easy to understand and context-appropriate, addressing their most troubling symptoms, such as pain, as well as what participants perceived as often-overlooked mental health concerns. Although the symptom and outcome items were easy to understand, the process of providing numeric ratings was unfamiliar and reported as difficult by some participants (Quote 4 in Table 3). However, participants reported confidence that data gathered via the mobile app would be accessed and used by clinical teams. This was reinforced when submitted reports were referred to by clinicians in subsequent meetings when discussing symptom management (Quote 5 in Table 3).

#### Theme 3: Impact of the Intervention on Health and Care

Some participants reported the intervention made respondents feel closer and more connected to their health care teams, making them feel more comfortable sharing symptoms and concerns with them (Quote 6 in Table 3). Participants also felt the app made the clinicians more knowledgeable about them as individuals, as well as their unique conditions. This was perceived as translating to quicker decision-making and improved access to medication and treatment (Quotes 7 and 8 in Table 3). The use of the app also contributed to patients’ agency, as they felt better informed and empowered to self-manage symptoms and to communicate the same with caregivers, family members, and the hospital team, where necessary. Some participants reported beneficial outcomes from reviewing and applying the self-management guidance (Quote 9 in Table 3). The intervention was found to have supported a focus on the mental health and well-being of participants. Some participants reported that having easily accessible information about their health condition helped to allay worries and anxieties and made them feel that help was available if required. The mobile phone app was reported to have fostered better interactions between patients, their caregivers and health workers, enabling them to recognize the need to seek help and receive it in a timely manner (Quote 10 in Table 3).

### Clinical Team Interview Findings

#### Theme 1: Perception of Impact of the Intervention on Patient Care

All participants from clinical teams reported that the intervention was beneficial to patient care. Participants reported feeling more connected with patients and valued the ability to assess changes promptly in a patient’s condition that could enable a timely response (Quote 11 in Table 3). However, the routine reporting by VHTs for some participants meant feeling more detached from patients, having instead to rely on reports provided by VHTs (Quote 12 in Table 3).

#### Theme 2: Experiences of Implementation

A period of adaptation was required for VHTs when using the mobile phone app, particularly for determining appropriate words for translating symptom and outcome items into local languages (Quote 13 in Table 3). This was overcome in collaboration with colleagues, with increased understanding of items by patients. The platform experienced some technical challenges during the study, with some participants reporting that reports submitted to the platform could not be viewed (Quote 14 in Table 3). In some instances, this led to delays and extra costs relating to patient care, as the VHTs often had to be called in to verify information or repeat the visit to the patient.

#### Theme 3: Data Robustness and Reporting

The clinical team members reviewing reports felt that additional options could be added to indicate which action was taken in response to reviewing a report. This included options such as being able to capture interaction or activity with other service providers. This was seen as a way of improving the usefulness of the dashboard (Quote 15 in Table 3). Some participants queried the wider use and linkage of data with existing government health information systems. Future integration with district health information systems was also viewed as useful, enabling the integration of palliative care data in existing processes for the Ministry of Health of Uganda (Quote 16 in Table 3). Alongside considering integration, a few participants raised concerns about data privacy and the safeguarding of personal data.

## Discussion

### Principal Findings

The findings from this study highlight the feasibility and acceptability of mPallCare, a digital health intervention for symptom monitoring and outcome reporting among patients accessing palliative care in a refugee settlement in Uganda. Initial high engagement with symptom and outcome reporting occurred, though engagement declined modestly over the study period. Initial challenges relating to navigation and translation of content, intermittent technical issues, connectivity limitations and competing community health duties may have contributed to this decline. Commonly reported symptoms included headaches, muscle pain, and dizziness. While there was varied completion of outcome assessments, overall trends for the study cohort suggested improvements in outcomes relating to pain, ability to share feelings, life feeling worthwhile, feeling at peace (ie, an item relating to aspects of perception of self and world, relationship to others and spiritual beliefs) and accessing help and advice. Patients reported that the intervention fostered a stronger connection with VHT volunteers and clinical teams, improved symptom management, and enhanced patient empowerment. However, challenges such as initial difficulties with navigating the content of the mobile phone app, translation issues, and occasional technical problems—most commonly data synchronization errors between the VHT app and the clinician dashboard—were identified. Health care professionals reported that the intervention facilitated more timely clinical decision-making, though reliance on intermediary reporting by VHTs sometimes limited direct patient interaction. The initial exploration of the feasibility and acceptability of the intervention suggests that it may have the potential to improve patient-centered care, but considerations for accessibility, training, and integration with existing health services remain crucial. While there were no clear differences in feasibility or overall acceptability across patient subgroups, variations in experience were noted between clinical roles. For example, nurses emphasized how reliance on intermediary reporting by VHTs occasionally limited direct engagement with patients, while VHTs described increased confidence and improved communication skills over time. These differences suggest the importance of exploring the delineation of complementary roles and feedback mechanisms between VHTs and clinical staff during future implementation.

In supporting the systematic collection of structured, patient-reported data, the intensive collection of longitudinal data by mPallCare can help to determine the temporal progression of patient-reported outcomes and better address individuals’ changing needs. For example, data can directly inform decision-making by care teams, alongside supporting preparation for community visits (eg, ensuring adequate medical supplies to support identified issues with symptom management). The approach also provides a means of undertaking both comparative effectiveness research and quality assessment relating to service delivery. This aligns with the research imperative to develop the evidence base underpinning care delivery in the context of humanitarian emergencies and crises [39] and builds on emerging applications of digital technologies, such as virtual training for palliative care [6] and more general development of approaches, including electronic health records [40]. Routinely captured data, such as symptom and outcomes data gathered via mPallCare, also provides opportunities to support policymaker decision-making, providing quality and timely data that can inform planning, decision-making and resource allocation in low-resource settings [41,42]. Investment in digital approaches such as mPallCare can support the collection of useful, relevant and reliable data that can help to characterize the current delivery of palliative care and guide the development of effective health services [43] across humanitarian emergencies and crises. This can ensure that the delivery of care is data-driven and reflects the needs of the population [44].

Having established the feasibility and acceptability of mPallCare, the platform is currently being evaluated across 20 additional clinical teams in Uganda and additional countries in Africa, including both refugee settlements and clinical care facilities outside refugee settlements. The initial development and testing of mPallCare reported in this manuscript highlighted multiple technical and user considerations for its future development. The design and accessibility of the intervention continue to be refined, including increasing the number of languages available (including local languages such as Bari, Ma’di, and Lingala, which have been incorporated) and the ease with which additional languages can be added. Such consideration of the accessibility and contextual relevance of a digital health intervention is essential to facilitate user engagement [45]. The decline in outcome reporting, alongside a slight increase in reported outcomes for most items, requires further research. The decline in outcome reporting over the 6-week study period warrants further reflection. Several factors may have contributed to this pattern. First, disease progression and declining physical function among participants with advanced illness likely limited their ability to engage consistently toward later data collection points. Similar declines in engagement have been observed in other mobile health interventions for people with serious illness, where symptom burden and reduced energy constrain sustained participation [35,46]. Second, competing priorities and workload among VHT members, who were responsible for data collection across multiple community programs, may have reduced the frequency of visits and the submission of reports. Third, technology fatigue and waning motivation once the initial novelty subsides—commonly noted in digital health studies—may have contributed to attrition, especially where perceived direct benefits to users were limited [47,48]. Intermittent network connectivity and temporary dashboard access problems also presented practical barriers. These findings mirror broader evidence from low-resource and humanitarian settings, where infrastructure limitations, connectivity challenges, and high clinical workloads hinder sustained digital engagement [37,49]. Future iterations of mPallCare will explore ways to mitigate these barriers, such as streamlined reporting schedules, automated reminders, stronger feedback mechanisms between clinicians and community teams, and increased user feedback on clinical action taken in response to submitted data.

In terms of the observed upward trends in Feelings, Peace, and Life, formal minimal clinically important differences (MCIDs) for the APCA POS have not been established. The palliative patient-reported outcome measures literature recommends interpreting change using anchor- and distribution-based approaches and explicitly estimating MCIDs for context-specific use [26,50,51]. Recent work for related instruments (eg, the Integrated Palliative Care Outcome Scale) is beginning to define MCIDs at the domain level (eg, dyspnea [52]) but comparable thresholds are not yet available for APCA POS. Accordingly, our findings should be interpreted as directional evidence consistent with expected benefits of structured symptom and outcome monitoring and regular follow-up—namely, improvements in psychological and existential well-being—rather than definitive proof of clinically important change. Future studies could prespecify MCID estimation for APCA POS (eg, using patient global impression of change anchors or distribution-based rules such as the SD of baseline or change scores), to enable a formal determination of clinical significance in humanitarian and low-resource settings.

Sustainability of digital health interventions in humanitarian settings remains a key challenge. The long-term viability of mPallCare may depend on factors critical to scale-up and sustainability, including ongoing technical support, training of community health workers, reliable internet access, and integration with national information systems to ensure interoperability and avoid duplication [53]. Sustaining engagement also requires ongoing supervision and feedback loops between VHTs and clinical teams, supported by consistent funding for device upkeep, data storage, and connectivity. These resource requirements mirror lessons from other digital health programs, where interventions that became embedded within Ministry of Health systems, supported by local champions and recurrent budget lines, were most likely to persist beyond pilot phases [54]. Successful integration will further depend on addressing key operational enablers and constraints. These include ensuring interoperability with existing digital systems such as District Health Information System 2 (DHIS2), maintaining compliance with national data protection policies, and sustaining Ministry of Health engagement for oversight and governance. Establishing clear data sharing agreements and local technical stewardship will be essential for scaling mPallCare responsibly and sustainably. In Uganda, mPallCare continues to be developed in partnership with the Ministry of Health of Uganda to ensure alignment with priorities for palliative care and refugee health, while exploring routes to integrate mPallCare with existing health information systems. This reflects both stakeholder enthusiasm and a feasible next step identified during qualitative interviews, where several clinical participants suggested integration with the national DHIS2 to strengthen data use and visibility of palliative care within national reporting structures. Furthermore, future work will evaluate the impact of mPallCare on patient-level outcomes, including symptom burden, quality of life, and timeliness of clinical review. This may require an approach such as a pragmatic, longitudinal mixed methods study design that incorporates repeated measures of patient-reported outcomes and clinician response times, with potential progression to a cluster randomized controlled trial following optimization.

Although evidence on digital health to support palliative care in humanitarian settings remains limited, our findings resonate with emerging work demonstrating how technology can strengthen communication and continuity of care in crisis contexts. In Lebanon, a mobile health tool for noncommunicable disease management among Syrian refugees improved documentation and patient satisfaction, though uptake was constrained by fragmented systems and parallel reporting [55]. Similarly, virtual learning networks have been found to build palliative care capacity among humanitarian health workers in Bangladesh, yet implementation was hindered by unstable connectivity and high staff turnover [6]. These studies, like the present one, highlight both the promise and fragility of digital innovation in settings characterized by workforce pressures and infrastructure limitations. This tension extends to telemedicine initiatives [56], which can be constrained by ad hoc funding and weak evaluation frameworks; yet evaluation is essential to avoid entrenching inequities [57]. mPallCare contributes to this body of evidence, illustrating that digital platforms can feasibly support palliative care when coupled with community engagement and integration within existing systems. Sustainability, however, remains central as most digital health projects undertaken in low- and middle-income countries falter beyond pilot phases [53]. For mPallCare, longevity may depend on embedding within national infrastructures such as DHIS2, maintaining technical and workforce capacity, and ensuring stable financing for maintenance and connectivity.

As mPallCare continues to develop, ethical considerations will be central to sustainability given its role in the context of often severe resource constraints. Routine symptom and outcome monitoring may highlight numerous issues for which a clear service response is not available. For example, outcome items relating to advice, concerns and being at peace were all scored relatively low by participants, yet there are many challenges in responding to these issues, such as accessing psychological support in the context of refugee settlements [58]. This creates a moral tension between the humanitarian principles of beneficence and nonmaleficence in resource-constrained contexts, where raising awareness of unmet need risks compounding distress if appropriate support cannot be mobilized [8,11]. During the study, interim mitigation was achieved through escalation pathways: VHTs could refer urgent clinical issues directly to the regional palliative care nurse, who provided intermittent outreach and psychosocial support. However, these mechanisms were limited by staffing and distance, highlighting the structural constraints of delivering equitable palliative care in humanitarian settings [11]. Future phases of mPallCare may address this ethical challenge through aligning system-level digital monitoring and response capacity. This could include, for example, developing simplified triage algorithms that prioritize symptoms or concerns requiring immediate action. Embedding such approaches within broader capacity-building efforts—particularly training frontline workers in basic psychological first aid and ensuring referral linkages for complex cases—will be essential to ensure that data collection translates into meaningful and safe care responses. Recognizing such ethical tensions, strategies to mitigate unmet needs are being incorporated into ongoing work. These include establishing partnerships with existing psychosocial and mental health programs operating within refugee settlements, embedding referral pathways into the digital platform, and curating evidence-informed digital self-help materials accessible directly through the mobile app. In addition, collaboration with the Ministry of Health aims to ensure that symptom and outcome data inform broader service planning, enabling scarce resources to be targeted toward the most pressing needs. Such measures seek to balance the ethical imperative of monitoring suffering with a commitment to providing or facilitating meaningful forms of support.

Implementation strategies and their evaluation form an essential element of digital health intervention development [59]. Continued evaluation to optimize how mPallCare can influence service responses within highly constrained resources will be essential to optimize its use in care delivery, alongside further understanding its role in enabling access to contextually appropriate self-management support. Additionally, the influence of different models of care delivery, staffing, local and broader policies, and case mix of patients beyond people living with advanced cancer can be explored through future scale-up and evaluation. Findings from Bidibidi Refugee Settlement provide valuable insights, but they will not be directly generalizable to all humanitarian settings. Contextual factors such as population stability, digital infrastructure, language diversity, and existing palliative care provision will influence implementation elsewhere. Nevertheless, the core principles of participatory design, health worker–led data collection, and integration with existing health systems may be broadly transferable to other displacement contexts. Furthermore, this is a small, uncontrolled feasibility study conducted over a 6-week period; findings are descriptive and not intended to infer efficacy. The limited sample size and reliance on VHT-facilitated reporting may have influenced patient responses. Furthermore, we used an uncontrolled, 6-week feasibility study, where findings are descriptive and not intended to infer efficacy or long-term sustainability. The short follow-up period and absence of a control group limit the generalizability of results beyond the study setting and timeframe. Future research using longitudinal or controlled designs will be required to evaluate sustainability and clinical impact. Despite these limitations, the study provides essential early evidence to guide refinement and larger-scale evaluation of mPallCare.

### Conclusion

This study underscores the feasibility of mPallCare, a palliative care–focused digital health intervention evaluated in the context of a refugee settlement in a low-resource setting. Routine symptom and outcome data collection was feasible, and key areas for further development were identified. The accessibility of mPallCare continues to be developed, including adding additional languages and planning for integration with existing health information systems. While this initial evaluation suggests mPallCare may foster improved patient-provider communication and support symptom management, challenges such as declining engagement over time, technical issues, and balancing the roles of VHTs and clinical teams must be addressed to optimize its implementation in clinical care. Ongoing adaptation and refinement will be critical to ensure mPallCare can support care delivery across diverse humanitarian and low-resource settings. Future research should continue to ensure continued evaluation throughout the ongoing scaling of mPallCare while maintaining a focus on usability and alignment with clinical workflows, including accommodating differing levels of staffing and resources to respond to identified symptoms and outcomes. The future success of mPallCare may be determined by its ability to support varied services and resource constraints, align with national health information systems, and effectively support patient-centered decision-making for palliative care delivery in the context of humanitarian and low-resource settings.

## Supplementary material

10.2196/73483 Multimedia Appendix 1 Symptom screening items used in mPallCare.

10.2196/73483 Checklist 1 COREQ checklist. 1

## Abbreviations

APCA: African Palliative Care Association
COREQ: Consolidated Criteria for Reporting Qualitative Research
DHIS2: District Health Information System 2
MCID: minimal clinically important difference
POS: Palliative Outcome Scale
VHT: village health team
