# Supplementary material for: Feasibility and Acceptability of mPallCare, a Digital Health Intervention for People Living With Advanced Cancer in a Refugee Settlement in Uganda: Mixed Methods Study
Source: JMIR Mhealth Uhealth. 2026 Jun 26;14:e73483. doi: 10.2196/73483 (PMC13308515; doi:10.2196/73483)
Supplement: Multimedia Appendix 1 [file mhealth-v14-e73483-s001.docx]

**Appendix A: Items included in the symptom screening tool**

| **Item** | | **Items indicative of tuberculosis** | **Items indicative of hepatitis B** | **Additional symptoms relevant to palliative care** |
| --- | --- | --- | --- | --- |
| Do you have a fever? | | X |  |  |
| Have you experienced any night sweats? | | X |  |  |
| Do you have a persistent cough (coughing a lot more than an hour, or 3 or more coughing episodes in 24 hours)? | | X |  |  |
| Have you been experiencing a prolonged cough of more than 2-3 weeks, with or without mucus that might be bloody? | | X |  |  |
| Have you experienced any unexplained weight loss? | | X |  |  |
| Have you experienced any clay-coloured stools in the last 24 hours? | |  | X |  |
| Have you experienced any yellowing of the skin? | |  | X |  |
| Over the last week have you experienced any of the following symptoms that continue to be troublesome? (please select all that apply): | |  |  |  |
|  | Headache |  |  | X |
|  | Sore or painful throat |  |  | X |
|  | Dizziness |  |  | X |
|  | Abdominal pain/stomach ache |  |  | X |
|  | Diarrhoea |  |  | X |
|  | Strong muscle pain |  |  | X |
|  | Red, itchy skin |  |  | X |
|  | Red sores or blisters on feet |  |  | X |
|  | Confusion, disorientation |  |  | X |
|  | Eye soreness or discomfort |  |  | X |
